# Association between Estrogen Receptor α Gene (*ESR1*) PvuII (C/T) and XbaI (A/G) Polymorphisms and Hip Fracture Risk: Evidence from a Meta-Analysis

**DOI:** 10.1371/journal.pone.0082806

**Published:** 2013-12-18

**Authors:** Li Tang, Guo-Lin Cheng, Zhong-Hua Xu

**Affiliations:** Department of Orthopedics, Jintan Hospital, Jiangsu University, Changzhou, China; Population Health and Preventive Medicine, Malaysia

## Abstract

**Background and Objective:**

Genetic factors are important in the pathogenesis of fractures. Notably, estrogen receptor α (*ESR1*) has been suggested as a possible candidate gene for hip fractures; however, published studies of *ESR1* gene polymorphisms have been hampered by small sample sizes and inconclusive or ambiguous results. The aim of this meta-analysis is to investigate the associations between two novel common *ESR1* polymorphisms (intron 1 polymorphisms PvuII-rs2234693: C>T and XbaI-rs9340799: A>G) and hip fracture.

**Methods:**

Crude odds ratios (ORs) with 95% confidence intervals (CIs) were used to evaluate the strength of the association.

**Results:**

Five case-control and three cohort studies were assessed, including a total of 1,838 hip fracture cases and 14,972 healthy controls. This meta-analysis revealed that the PvuII T allele is a highly significant risk factor for hip fracture susceptibility, with an effect magnitude similar in male and pre-menopausal and post-menopausal female patients. In stratified analysis based on ethnicity, the PvuII T allele remained significantly correlated with increased risk of hip fracture in Caucasian populations; this correlation, however, was not found in Asian populations. Unlike the PvuII polymorphism, we did not find significant differences in the XbaI (A>G) polymorphism allele or genotype distributions of hip fracture patients and controls. We also found no obvious association between the XbaI polymorphism and hip fracture in any of the racial or gender subgroups.

**Conclusion:**

Our findings show that the *ESR1* PvuII T allele may increase the risk of hip fracture and that the XbaI polymorphism is not associated with hip fracture.

## Introduction

Hip fracture, the most serious complication of osteoporosis, is a common musculoskeletal disorder [1]. It accounts for the majority of fracture related health care expenditure and mortality in men and women over the age of 50 years worldwide [2], [3]. The global annual rate of hip fracture has been exponentially increasing, with a current rate of approximately 1.5 million cases per year [4], [5]. In addition, with growing aging populations, the number of osteoporosis cases is expected to increase to 2.6 million by 2025 and to 4.5 million by 2050 [6]. Recently, numerous studies have attempted to explore the pathogenesis of this disease [7−10]. Bone mineral density (BMD) has been found to be an important clinical predictor of fracture risk. Most variance in BMD could be due to genetic factors, with as much as 65−92% of the difference in BMD attributable to genetic influences [11], [12]. Rapid progress has been made in recent years to identify the genes and alleles that affect hip fracture risk, such as the vitamin D receptor (*VDR*) gene, insulin-like growth factor I (*IGF-I*) gene, collagen type I alpha 1(*COL1A1*) gene and estrogen receptor α gene (*ESR1*), etc [13−15].

Estrogen receptor α, a member of the nuclear receptor super-family of ligand-activated transcription factors, is one of the key mediators of hormonal response in estrogen-sensitive tissues [16], [17]. The estrogen-ESR1 complex is primarily responsible for regulating cellular signal pathways in vivo, as well as bone mass in skeletal systems [18], [19]. It has been shown that serum estradiol level may be a predictor of subsequent bone mass density [20], [21] and risk for osteoporotic fractures [22], [23]. Based on the observation that osteoporotic fractures are prevalent among women, and especially among postmenopausal women, estrogen is also believed to play an important role in hip fractures [24]. Thus, genetic variations in *ESR1,* which are caused by alternative splicing that alters the expression of ESR1, are likely to affect hip fracture susceptibility [25]. Several genetic polymorphisms of the *ESR1* gene, including *ESR1* XbaI (rs9340799, A/G) and PvuII (rs2234693, C/T), have been investigated for a possible association with hip fracture risk [26−28]. Two recent genome wide association studies (GWAS) have found that several loci in the 6q25 *ESR*1 region are associated with bone mineral density of the hip and spine, providing rapid insights into the genetic association between ESR1 gene and hip fracture risk [29], [30]. A previous meta-analysis by Ioannidis et al. found that the adjusted odds of vertebral fracture were reduced by 35% in women homozygous for the absence of an XbaI recognition site, while no significant effects on fracture risk were observed for PvuII polymorphism [31]. However, the results from another recent meta-analysis by Lei et al. indicated that a modest, but statistically significant, association between the *ESR1* PvuII pp genotype and vertebral fracture existed in five case-control studies, but no association between the PvuII polymorphism and hip fracture were observed in one study [32].

Two previous meta-analyses have concluded that the *ESR1* XbaI polymorphism may be associated with decreased risk of vertebral fracture in women and that the PvuII polymorphism is not associated with vertebral fracture risk [31], [32]; however, they did not provide evidence of the two polymorphisms’ correlation with hip fracture susceptibility. Gender differences are extensively reported in the epidemiology of hip fracture, which is particularly prevalent among postmenopausal women. Therefore, we performed a meta-analysis of all eligible case-control studies associating hip fracture risk linked with gender and menopausal status in order to explicate the relationship between two common polymorphisms (PvuII and XbaI) in the *ESR1* gene and hip fracture susceptibility. We hope this meta-analysis will help with the early identification and therapeutic treatment of hip fracture.

## Materials and Methods

### Literature search

We performed an electronic search for papers published before June 19th, 2013 in PubMed, Embase, Web of Science, Chinese National Knowledge Infrastructure (CNKI), and Chinese Biomedical Literature Database (CBM). Literature searches were performed by an expert using the following key words and MeSH terms: (‘fractures, hip’ or ‘subtrochanteric fractures’ or ‘femoral neck fractures’ or ‘femur neck fractures’), (‘genetic polymorphism’ or ‘single nucleotide polymorphisms’ or ‘gene mutation’ or ‘genetic variants’) and (‘estradiol receptor alpha’ or ‘ER alpha’ or ‘estrogen receptor 1’ or ‘*ESR1*’). The reference lists of the prospective articles were also reviewed to identify additional relevant publications and studies.

### Inclusion and exclusion criteria

Studies included in our meta-analysis had to meet the following criteria: (a) population-based case-control, cohort or cross-sectional study focusing on associations of *ESR1* PvuII and/or XbaI polymorphisms with hip fracture risk; (b) hip fracture cases must be diagnosed as pathologic fractures or fractures resulting from trauma other than a fall; (c) inclusion of sufficient data on sample size, odds ratio (OR), and 95% confidence interval (CI); and (d) published in the English or Chinese language.

Studies were excluded when they represented duplicates of previous publications, or were meta-analyses, letters, reviews or editorial articles. Additionally, when data were included in multiple studies using the same case series, either the study with the largest sample size or the study with the most recently publication date was selected. All disagreements on study inclusion were resolved through discussions. To ensure the rigor of the current meta-analysis, we designed and reported the meta-analysis according to the Preferred Reporting Items for Systematic Reviews and Meta-analyses (PRISMA) statement. The relevant checklist is shown in Supplement S1.

### Data extraction

All data from included studies were extracted independently by two investigators using a piloted data standardized form (any discrepancies were resolved through discussion and, when necessary, adjudicated by a third reviewer): the first author’s surname, year of publication, country of origin, published language, gender and ethnicity of study subjects, study design, number of subjects, SNP genotyping methods, genotyping method and detected sample, allele and genotype frequencies, and evidence of Hardy-Weinberg equilibrium (HWE) in controls. In addition, we also compared key study characteristics, such as location, publication date and authorship, to determine the existence of multiple publications from the same study.

### Quality assessment of included studies

Two authors independently assessed the quality of the published articles according to the modified STROBE quality score systems [33]. Forty assessment items matching with the quality appraisals were used in this meta-analysis, with scores ranging from 0 to 40. Scores of 0−20, 20−30 and 30−40 were defined as low, moderate and high quality, respectively. Differences were resolved through discussions between the two authors; if no agreement could be reached, a third reviewer was consulted. The modified STROBE quality score system is available in Supplement S2.

### Statistical analysis

Crude ORs together with their corresponding 95% CIs were used to calculate and assess the strength of associations between *ESR1* PvuII and/or XbaI polymorphisms and hip fracture risk under five genetic models: allele, dominant, recessive, homozygous, and heterozygous models. The deviation of frequency from those expected under Hardy-Weinberg equilibrium (HWE) was assessed by Chi-squared goodness of fit tests in the controls. We explored inter-study variation by prespecified subgrouping of studies according to ethnicity (Caucasian, Asian), gender (female, male) and menopausal status among women (pre-menopausal, post-menopausal), where applicable. The statistical significance of the pooled OR was assessed with a Z test. Between-study variation and heterogeneity were estimated using Cochran’s *Q*-statistic, with *P*<0.05 as a cutoff for statistically significant heterogeneity [34].

We also quantified the effect of heterogeneity with the *I^2^* test (ranges from 0 to 100%), which represents the proportion of inter-study variability that can be attributed to heterogeneity rather than to chance [35]. The fixed effects model (*Mantel-Haenszel* method) was used, except when a significant *Q*-test (*P*<0.05) or *I^2^*>50% indicated the existence of heterogeneity among studies; otherwise, the random effects model (*DerSimonian-Laird* method) was applied to the meta-analysis. In order to ensure the reliability of results, a sensitivity analysis was performed by omitting individual studies. Begger’s funnel plots were used to detect publication bias. In addition, Egger’s linear regression test, which measures funnel plot asymmetry via a natural logarithm scale of OR, was also used to evaluate publication bias [36]. All *P*-values were two-sided. Analyses were conducted with STATA Version 12.0 software (Stata Corp, College Station, TX). Meta analysis, sensitivity analysis and publication bias were conducted using the following STATA’s user-written functions: metan, metaninf and metabias, respectively.

## Results

### The characteristics of included studies

**Figure 1:**
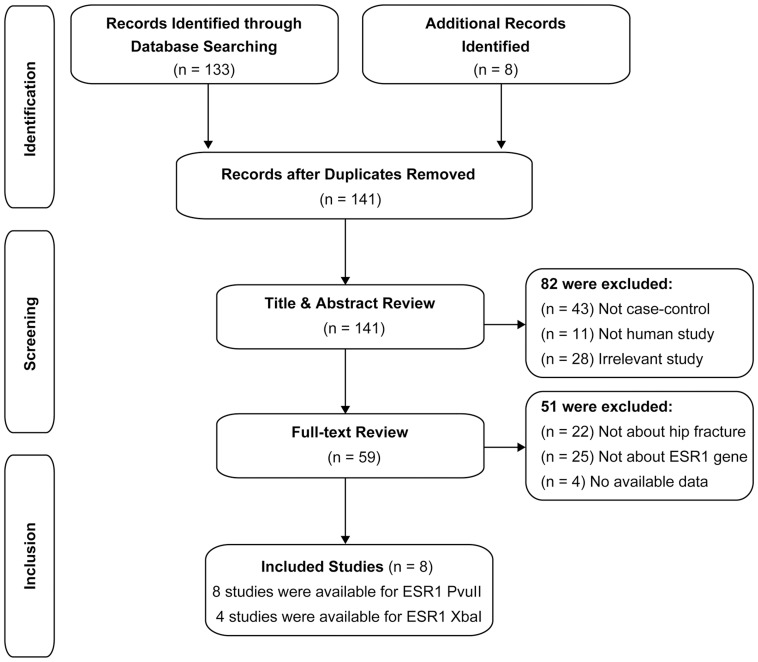
Flow diagram of selection of studies and specific reasons for exclusion from the present meta-analysis.

Literature search yielded 129 reports, of which five population-based case-control [25], [26], and three cohort [27], [40], [41] studies met the inclusion criteria for studies on the associations between hip fracture susceptibility and the two common polymorphisms of *ESR1*, PvuII (rs2234693) and XbaI (rs9340799) in intron 1. A flow diagram of the studies selection process, as well as the specific reasons for exclusion from the meta-analysis is shown in Figure 1. The selection process involved three steps: identification, screening and inclusion. 141 relevant reports were initially indentified, 82 were excluded after title and abstract review and 51 more were excluded after full-text review. Finally, eight studies were included in this meta-analysis. The publication years of the selected studies ranged from 2000 to 2012. There were six studies on subjects of Caucasian descent and two studies on subjects of Asian descent. All included studies extracted DNA from peripheral blood and a classic PCR-RFLP assay was used in 6 out of 8 studies. Genotype frequencies of healthy controls in all studies were consistent with the HWE test. The qualities of the included studies were moderately high with a STROBE score greater than 20. The detailed characteristics of the involved studies are listed in Table 1. The genotype distribution and allele frequency data is shown in Supplement S3.

**Table 1:** Main characteristics and methodological quality of all eligible studies.

First author	Year	Country	Ethnicity	Simple size	Enrolled	Study	Genotype	Mutation	MAF	HWE	Association	Outcome	Quality
				(case/control)	patient	type	method	site	(case/control)	*P* value	*P* value		score
Wei et al	2012	China	Asian	60/120	HF	PCC	PCR-RFLP	PvuII	0.48/0.51	0.71	*P* = 0.66	NS	23/40
Wang et al	2012	China	Asian	128/128	OHF	PCC	PCR-RFLP	PvuII	0.71/0.60	0.17	*P* = 0.007	SA	21/40
								XbaI	0.73/0.77	0.19	*P* = 0.026	NS	
Valero et al	2008	Spain	Caucasian	489/356	HF	PCC	Taqman	PvuII	0.54/0.51	0.34	*P* = 0.39	NS	24/40
Massart et al	2009	Italy	Caucasian	102/725	HF	Cohort	PCR-RFLP	PvuII	0.58/0.52	0.57	*P*<0.01	SA	25/40
				61/279	OHF	Cohort	PCR-RFLP	PvuII	0.59/0.51	1.00	*P*<0.01	SA	
Lian et al	2007	USA	Caucasian	569/4134	OHF	Cohort	PCR-RFLP	PvuII	0.58/0.53	0.53	*P* = 0.01	SA	23/40
								XbaI	0.67/0.65	0.10	*P* = 0.19	NS	
Kjaergaard et al	2007	Denmark	Caucasian	179/4938	HF in women	Cohort	PCR-RFLP	PvuII	0.56/0.54	0.40	NA	NS	24/40
				94/4032	HF in men	Cohort	PCR-RFLP	PvuII	0.62/0.55	0.63	NA	NS	
Dincel et al	2008	Turkish	Caucasian	21/21	OHF	PCC	AS-PCR	PvuII	0.50/0.40	0.46	NA	Unstated	22/40
								XbaI	0.50/0.34	0.43	NA	Unstated	
Aerssens et al	2000	Belgium	Caucasian	135/239	OHF	PCC	PCR-RFLP	PvuII	0.56/0.54	0.41	*P* = 0.79	NS	27/40
								XbaI	0.66/0.63	0.99	*P* = 0.69	NS	

HF  =  hip fracture, OHF  =  osteoporotic hip fracture, PCC  =  population-based case-control study, PCR-RFLP  =  Polymerase chain reaction restriction fragment length polymorphism, AS-PCR  =  allele-specific polymerase chain reaction, MAF  =  minor allele frequency, HWE  =  Hardy-Weinberg equilibrium, NA  =  not available, NS  =  not significant, SA  =  significant association.

### Association between *ESR1* PvuII (C>T) polymorphism and hip fracture risk

The association of the *ESR1* PvuII (C>T) polymorphism and hip fracture was investigated in eight studies, with a total of 1,838 cases and 14,972 healthy controls. Among these studies, six were conducted in Caucasian populations and two in Asian populations. The findings of this meta-analysis on the correlation between the polymorphism and hip fracture risk are summarized in Table 2.

**Table 2:** Subgroup analyses for the associations of ESR1 PvuII (C/T) with hip fracture risk.

Subgroups	No. of case/control	Allele model		Dominant model		Recessive model		Homozygous model		Heterozygous model	
		(T allele vs. C allele)		(TT + CT vs. CC)		(TT vs. CC + CT)		(TT vs. CC)		(TT vs. CT)	
		OR (95%CI)	*P* value	OR (95%CI)	*P* value	OR (95%CI)	*P* value	OR (95%CI)	*P* value	OR (95%CI)	*P* value
***Ethnicity***											
**Asian**	188/248	1.24 (0.69−2.24)	0.478	1.17 (0.69−2.00)	0.559	1.43 (0.65−3.14)	0.374	1.47 (0.80−2.69)	0.216	1.45 (0.74−2.86)	0.281
**Caucasian**	1655/14517	1.18 (1.09−1.28)	<0.001	1.26 (1.09−1.45)	0.002	1.24 (1.10−1.40)	0.001	1.39 (1.18−1.64)	<0.001	1.18 (1.04−1.34)	0.013
***Gender***											
**Male**	222/4254	1.46 (1.15−1.83)	0.002	1.80 (1.10−2.94)	0.019	1.59 (1.16−2.20)	0.005	2.16 (1.27−3.67)	0.004	1.44 (1.03−2.20)	0.033
**Female**	1542/10371	1.17 (1.07−1.29)	<0.001	1.22 (1.05−1.42)	0.001	1.23 (1.08−1.39)	0.002	1.35 (1.14−1.61)	0.001	1.18 (1.03−1.35)	0.017
***Menopausal status***											
**Pre-menopausal**	163/326	1.30 (1.02−1.66)	0.033	1.49 (1.04−1.43)	0.079	1.38 (0.96−1.99)	0.081	1.73 (1.04−2.88)	0.033	1.27 (0.86−1.87)	0.226
**Post-menopausal**	1260/4828	1.15 (1.04−1.26)	0.005	1.18 (1.00−1.40)	0.054	1.21 (1.05−1.41)	0.010	1.31 (1.08−1.59)	0.007	1.18 (1.00−1.37)	0.043
***Genotype methods***											
**PCR-RFLP**	1053/5325	1.20 (1.09−1.32)	<0.001	1.24 (1.04−1.49)	0.016	1.31 (1.13−1.51)	<0.001	1.42 (1.16−1.74)	0.001	1.26 (1.08−1.47)	0.004
**TaqMan**	771/9420	1.16 (1.02−1.32)	0.024	1.25 (1.00−1.57)	0.050	1.19 (0.98−1.44)	0.087	1.35 (1.04−1.75)	0.024	1.12 (0.91−1.38)	0.273
**AS-PCR**	19/20	1.50 (0.61−3.68)	0.376	1.87 (0.48−7.26)	0.368	1.43 (0.32−6.39)	0.641	2.00 (0.36−11.2)	0.431	1.11 (0.22−5.63)	0.899
**Overall**	1843/14765	1.19 (1.10−1.28)	<0.001	1.25 (1.09−1.44)	0.001	1.26 (1.12−1.42)	<0.001	1.40 (1.19−1.64)	<0.001	1.21 (1.07−1.36)	0.003

OR  =  odds ratio; CI  =  confidence intervals; PCR-RFLP  =  Polymerase chain reaction restriction fragment length polymorphism; AS-PCR  =  allele-specific polymerase chain reaction.

**Figure 2:**
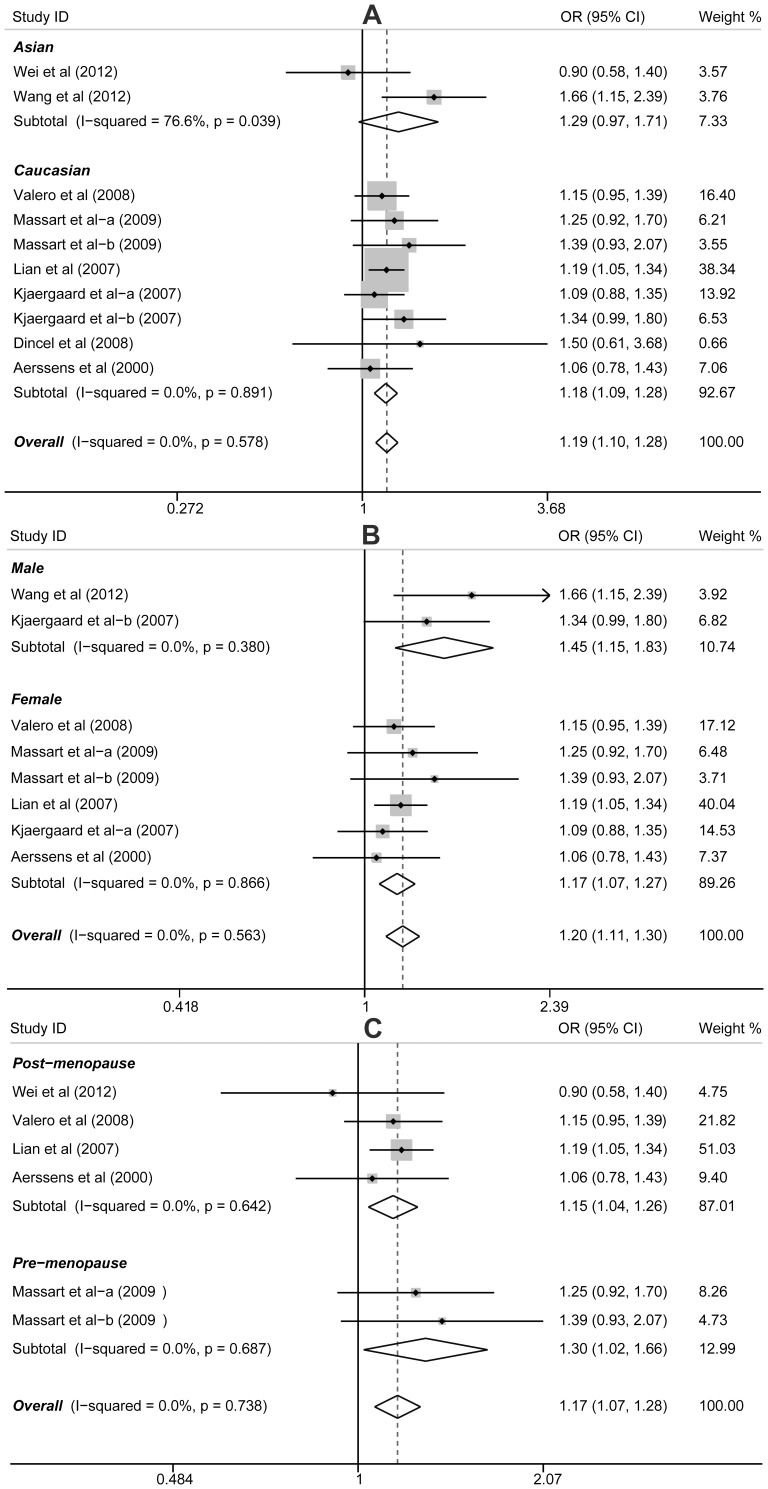
Forest plot of ORs for the association between PvuII (C>T) polymorphism and susceptibility to hip fracture in subgroup analysis based on ethnicity (A), gender (B), and menopausal status (C) under the allele model.

The results revealed that a highly significant increased effect was conferred by the PvuII T allele on hip fracture risk, with an approximately 18% increment in the odds (for allele model: OR = 1.19, 95%CI: 1.10−1.28, *P*<0.001; dominant model: OR = 1.25, 95%CI: 1.09−1.44, *P* = 0.001; recessive model: OR = 1.26, 95%CI: 1.12−1.42, *P*<0.001; homozygous model: OR = 1.40, 95%CI: 1.19−1.64, *P*<0.001; heterozygous model: OR = 1.21, 95%CI: 1.07−1.36, *P* = 0.003), and the magnitude of the effect was similar in female (for allele model: OR = 1.17, 95%CI: 1.07−1.29, *P*<0.001; dominant model: OR = 1.22, 95%CI: 1.05−1.42, *P* = 0.001; recessive model: OR = 1.23, 95%CI: 1.08−1.39, *P* = 0.002; homozygous model: OR = 1.35, 95%CI: 1.14−1.61, *P* = 0.001; heterozygous model: OR = 1.18, 95%CI: 1.03−1.35, *P* = 0.017) and male (for allele model: OR = 1.46, 95%CI: 1.15−1.83, *P* = 0.002; dominant model: OR = 1.80, 95%CI: 1.10−2.94, *P* = 0.019; recessive model: OR = 1.59, 95%CI: 1.16−2.20, *P* = 0.005; homozygous model: OR = 2.16, 95%CI: 1.27−3.67, *P* = 0.004; heterozygous model: OR = 1.44, 95%CI: 1.03−2.20, *P* = 0.033) (Figure 2B). In addition, we isolated the study including pre-menopausal and post-menopausal women and analyzed the data in both groups. In premenopausal women, we observed that PvuII (C/T) is significantly correlated with increased risk of hip fracture under allele and homozygous models (OR = 1.30, 95%CI: 1.02−1.66, *P* = 0.033; OR = 1.73, 95%CI: 1.04−2.88, *P* = 0.033). In postmenopausal women, we also observed that TT genotype is associated with higher risk of hip fracture than either TC or CC subjects (for allele model: OR = 1.15, 95%CI: 1.04−1.26, *P* = 0.005; recessive model: OR = 1.21, 95%CI: 1.05−1.41, *P* = 0.010; homozygous model: OR = 1.31, 95%CI: 1.08−1.59, *P* = 0.007; heterozygous model: OR = 1.18, 95%CI: 1.00−1.37, *P* = 0.043) (Figure 2C). Interestingly, in the stratified analysis by ethnicity, the PvuII T allele is significantly correlated with increased risk of hip fracture among Caucasian populations (for allele model: OR = 1.18, 95%CI: 1.09−1.28, *P*<0.001; dominant model: OR = 1.26, 95%CI: 1.09−1.45, *P* = 0.002; recessive model: OR = 1.24, 95%CI: 1.10−1.40, *P* = 0.001; homozygous model: OR = 1.39, 95%CI: 1.18−1.64, *P*<0.001; heterozygous model: OR = 1.18, 95%CI: 1.04−1.34, *P* = 0.013), while such result was not found in Asian populations (all *P*>0.05) (Figure 2A).

### Association between *ESR1* XbaI (A>G) polymorphism and hip fracture risk

**Figure 3:**
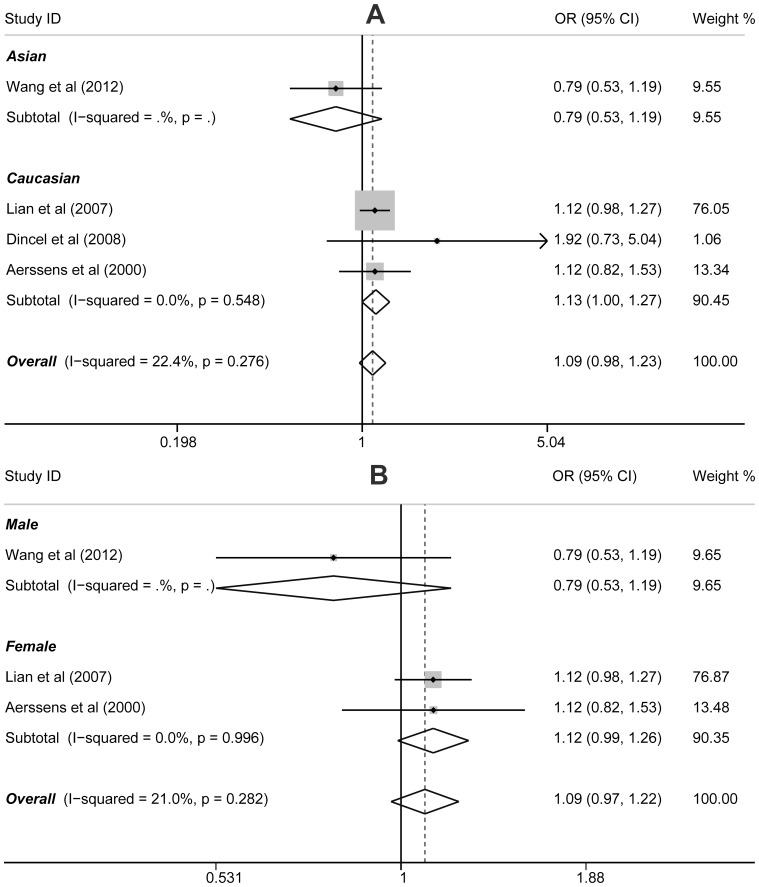
Forest plot of ORs for the association of XbaI (A>G) polymorphism and susceptibility to hip fracture in subgroup analysis based on ethnicity (A) and gender (B) under the allele model.

The association between the *ESR1* XbaI (A>G) polymorphism and hip fracture was investigated only in four studies, with a total of 853 cases and 4,522 healthy controls. Among these studies, three were on Caucasians and one on Asians. A summary of the meta-analysis findings on the association of the polymorphism with hip fracture risk is shown in Table 3. The initial meta-analysis showed that there was no statistically significant association between the XbaI polymorphism and hip fracture under any of the genetic models (for allele model: OR = 1.09, 95%CI: 0.97−1.23, *P* = 0.125; dominant model: OR = 1.14, 95%CI: 0.89−1.47, *P* = 0.290; recessive model: OR = 1.12, 95%CI: 0.96−1.30, *P* = 0.161; homozygous model: OR = 1.20, 95%CI: 0.93−1.57, *P* = 0.167; heterozygous model: OR = 1.10, 95%CI: 0.94−1.29, *P* = 0.245). The association between either ethnicity or menopausal status and the polymorphic genotypes were also not statistically significant for any of these analyses (all *P*>0.05, data not shown) (Figure 3).

**Table 3:** Subgroup analyses for the associations of ESR1 XbaI (A/G) with hip fracture risk.

Subgroups	No. of case/control	Allele model		Dominant model		Recessive model		Homozygous model		Heterozygous model	
		(G allele vs. A allele)		(GG + AG vs. AA)		(GG vs. AA + AG)		(GG vs. AA)		(GG vs. AG)	
		OR (95%CI)	*P* value	OR (95%CI)	*P* value	OR (95%CI)	*P* value	OR (95%CI)	*P* value	OR (95%CI)	*P* value
*Ethnicity*											
Asian	128/128	0.79 (0.53−1.19)	0.261	0.66 (0.18−2.38)	0.522	0.75 (0.46−1.23)	0.259	0.59 (0.16−2.17)	0.423	0.77 (0.47−1.28)	0.312
Caucasian	720/4381	1.13 (1.00−1.27)	0.053	1.17 (0.91−1.51)	0.230	1.17 (0.99−1.37)	0.064	1.24 (0.95−1.63)	0.117	1.15 (0.97−1.36)	0.116
*Gender*											
Male	128/128	0.79 (0.53−1.19)	0.261	0.66 (0.18−2.38)	0.552	0.75 (0.46−1.23)	0.259	0.59 (0.16−2.17)	0.423	0.77 (0.47−1.28)	0.312
Female	704/4362	1.12 (0.99−1.26)	0.075	1.14 (0.88−1.47)	0.329	1.16 (0.99−1.37)	0.073	1.22 (0.93−1.60)	0.154	1.15 (0.97−1.36)	0.118
*Genotype methods*											
PCR-RFLP	704/4362	1.08 (0.97−1.22)	0.166	1.11 (0.86−1.43)	0.404	1.11 (0.95−1.30)	0.179	1.18 (0.91−1.54)	0.215	1.10 (0.94−1.29)	0.249
AS-PCR	16/19	1.92 (0.73−5.04)	0.184	2.70 (0.64−11.5)	0.178	1.78 (0.33−9.49)	0.500	3.00 (0.45−20.1)	0.258	1.17 (0.19−7.12)	0.867
Overall	848/4509	1.09 (0.97−1.23)	0.125	1.14 (0.89−1.47)	0.290	1.12 (0.96−1.30)	0.161	1.20 (0.93−1.57)	0.167	1.10 (0.94−1.29)	0.245

OR = odds ratio; CI  =  confidence intervals; PCR-RFLP  =  Polymerase chain reaction restriction fragment length polymorphism; AS-PCR  =  allele-specific polymerase chain reaction.

### Sensitivity analysis and publication bias

**Figure 4:**
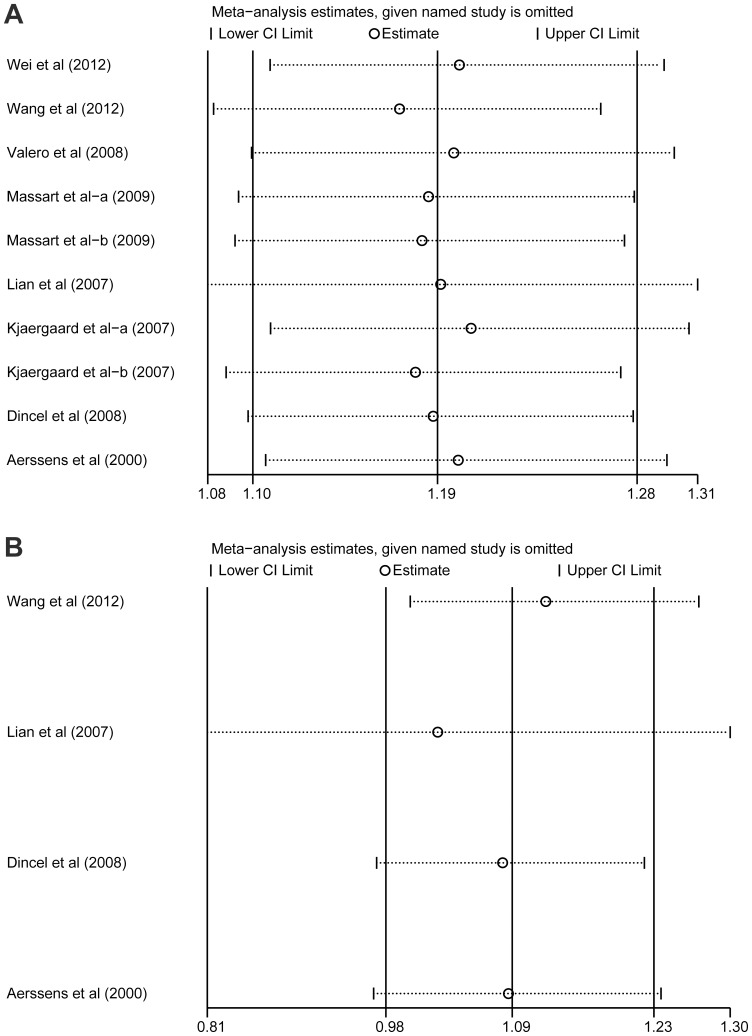
Sensitivity analysis of the summary odds ratio coefficients of PvuII (C>T) and XbaI (A>G) polymorphisms are illustrated under the allele model. Results were computed by omitting each study in turn. The two ends of the dotted lines represent the 95% CI (A: PvuII; B: XbaI).

Sensitivity analysis was performed to assess the influence of each study on the pooled ORs by omitting individual studies. The analysis results suggested that no individual study significantly altered the pooled ORs for either *ESR1* PvuII (C>T) or XbaI (A>G) under the allele model (Figure 4), indicating that our studies were statistically accurate.

**Figure 5:**
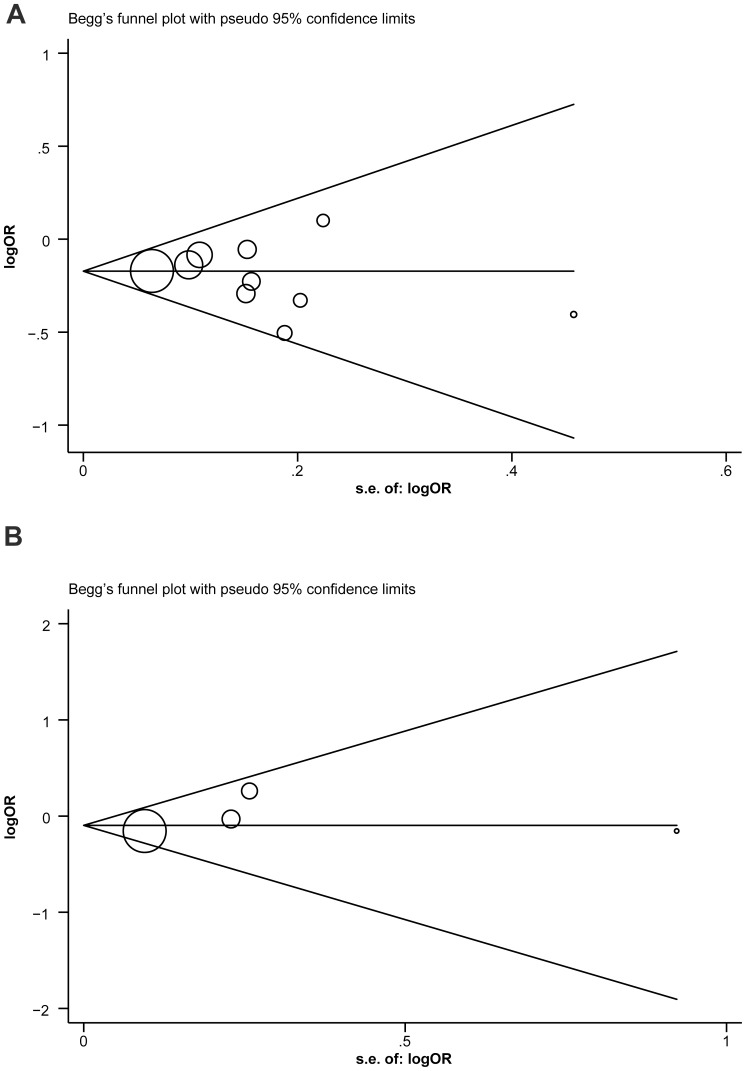
Begger’s funnel plot of publication bias in the selection of studies for PvuII (C>T) and XbaI (A>G) polymorphisms are illustrated (A: PvuII; B: XbaI). Each point represents a separate study by the indicated association. Log[OR], natural logarithm of OR. Horizontal line, mean magnitude of the effect.

Begger's funnel plot and Egger's linear regression test were performed on the metadata to assess the publication bias of individual studies. The shapes of the funnel plots did not reveal any evidence of obvious asymmetry for either the *ESR1* PvuII (C>T) or XbaI (A>G) (Figure 5). Egger's test also displayed no significant statistical evidence of publication bias (PvuII: t = −0.78, *P* = 0.456; XbaI: t = 0.96, *P* = 0.440).

## Discussion

Over the past decade, many linkage and association studies have been performed to find candidate genes in order to analyze the relationship between genetic factors and complex traits, such as BMD and/or fracture risk [42]. It was reported that small alterations in BMD could result in a significant difference in fracture risk [43]. Thus, many genes in each category were proposed for their association with normal BMD variation, yielding an ever expanding candidate gene list. One of the most widely studied is the *ESR1* gene [7]. A genome wide association study (GWAS) in 2008 reported that five SNPs (rs9479055, rs4870044, rs1038304, rs6929137, rs1999805) in the 6q25 *ESR1* region showed an association with bone mineral density of both the hip and spine, suggesting the possible role of the *ESR1* gene in the pathogenesis of osteoporosis [29]. A more recent GWAS further confirmed the association between the *ESR1* gene and osteoporotic fractures [30]. The most studied variants of the *ESR1* gene are the PvuII and XbaI polymorphisms, which have been linked to a lower sensitivity to estrogen [44−46]. However, the association between *ESR1* gene polymorphisms and fracture risk is still controversial and ambiguous. To date, there are only two published meta-analyses that evaluate the possibility of a significant association between the *ESR1* PvuII polymorphism and risk of fracture. The meta-analysis by Ioannidiset al. showed that the PvuII polymorphism was not associated with fracture risk [7], whereas the other one by Lei et al. suggested a modest but statistically significant association between the *ESR1* PvuII pp genotype and vertebral fracture [32]. Therefore, we decided to perform a meta-analysis of all eligible case-control studies on hip fracture risk in order to reveal a more accurate relationship between the PvuII and XbaI polymorphisms of the *ESR1* gene and risk of hip fracture.

This meta-analysis evaluated the association between two common *ESR1* polymorphisms and hip fracture. The results of this meta-analysis show that the PvuII polymorphism has a statistically significant association with hip fracture, especially in Caucasian populations but not in Asian populations. The discrepancy between ethnicity subgroups may be due to sample size since only 188 cases and 248 controls were studied in Asian populations. Other than family history and ethnicity, the most well-established risk factors for hip fracture are gender and menopausal status in females [47], [48]. Therefore, we performed a stratified analysis based on gender and menopausal status. Our data revealed that the PvuII T allele was a risk factor in the subgroup of premenopausal and postmenopausal women, and men. Unlike the PvuII polymorphism, no significant difference was found in the XbaI (A>G) polymorphism allele or genotype distribution between hip fracture patients and controls. However, this result warrants further investigations since only four studies with small sample sizes examined the XbaI (A>G) polymorphism. We also performed a stratified analysis based on ethnicity and gender for the *ESR1* XbaI (A>G) polymorphism. We found no obvious association between the XbaI polymorphism and hip fracture in either racial or gender subgroups. This discrepancy in the associations of the two polymorphisms may be due to sample size since there are only eight studies on PvuII and four on XbaI. Although these variants alone are not clinically useful in the prediction of risk to the individual person, their interactions with candidate genes identified by the GWAS may play important roles in the pathogenesis underlying hip fracture.

In interpreting the results of this meta-analysis, some specific issues should be mentioned. First, as with other complex traits, hip fracture risk may be modulated by several other genetic markers besides *ESR1* gene, including polymorphisms of the *VDR* gene, the *COL1A1* gene, the *IGF-I* gene, and several other candidate genes. Thus, our meta-analysis emphasizes that elucidating the pathogenesis of fracture demands an investigation into the association of many gene variants constituting distinct pathophysiological pathways. Second, we should emphasize that this meta-analysis does not address the question of whether the *ESR1* gene shows evidence of genetic linkage to BMD. Third, we identified only two studies from Asian populations and obtained no data from African populations, thus these two racial groups demand further studies. Last, this meta-analysis was based on unadjusted ORs estimates as a result of the lack of available information, preventing a more precise evaluation with adjusted ORs by certain covariates such as age, BMI, and smoking status, etc; thus, our data is only a conservative estimates of the association between *ESR1* gene and hip fracture. Ideally, our conclusions will be tested by future studies. Despite the limitations listed above, our meta-analysis has some strength. To the best of our knowledge, this is the first meta-analysis on the relationship between the *ESR1* gene polymorphisms and hip fracture. We explored inter-study variations by subgrouping studies according to ethnicity, gender and menopausal status for females. Furthermore, although this meta-analysis does not accommodate all previously published data, that is limited compared to the evidence here generated.

In summary, this meta-analysis of eight studies indicates that allelic variation in the PvuII (C>T) polymorphism of *ESR1* gene may be a risk factor for hip fracture, with a similar effect magnitude in both pre-menopausal and post-menopausal females, and male. However, unlike with the PvuII polymorphism, no significant difference was found in the XbaI (A>G) polymorphism allele or genotype distribution between hip fracture patients and controls, either in racial or gender subgroups. Thus, our results do support the hypothesis that the PvuII (C>T) polymorphism has potential clinical value as a predictors of hip fracture. Based on the limitations mentioned above, it is critical that large, well-designed studies are performed to re-evaluate the potential associations between *ESR1* gene polymorphisms with other candidate gene polymorphisms and hip fracture risk.

## Supporting Information

Supplement S1 **PRISMA Checklist**.
(DOC)

Supplement S2 **Modified STROBE quality score systems**.
(DOC)

Supplement S3 **The genotype distribution and allele frequencies of** ***ESR1*** **PvuII and XbaI polymorphisms**.
(XML)
